# The Functional Impact of Breast Reconstruction: An Overview and Update

**DOI:** 10.1097/GOX.0000000000001640

**Published:** 2018-03-06

**Authors:** Jonas A. Nelson, Iris T. Lee, Joseph J. Disa

**Affiliations:** From the * Section of Plastic and Reconstructive Surgery, Memorial Sloan Kettering Cancer Center, New York, N.Y.; and † Department of Obstetrics and Gynecology, Hospital of the University of Pennsylvania, Philadelphia, Pa.

## Abstract

As rates of bilateral mastectomy and immediate reconstruction rise, the aesthetic and psychosocial benefits of breast reconstruction are increasingly well understood. However, an understanding of functional outcome and its optimization is still lacking. This endpoint is critical to maximizing postoperative quality of life. All reconstructive modalities have possible functional consequences. Studies demonstrate that implant-based reconstruction impacts subjective movement, but patients’ day-to-day function may not be objectively hindered despite self-reported disability. For latissimus dorsi flap reconstruction, patients also report some dysfunction at the donor site, but this does not seem to result in significant, long-lasting limitation of daily activity. Athletic and other vigorous activities are most affected. For abdominal free flaps, patient perception of postoperative disability is generally not significant, despite the varying degrees of objective disadvantage that have been identified depending on the extent of rectus muscle sacrifice. With these functional repercussions in mind, a broader perspective on the attempt to ensure minimal functional decline after breast surgery should focus not only on surgical technique but also on postoperative rehabilitation. Early directed physical therapy may be an instrumental element in facilitating return to baseline function. With the patient’s optimal quality of life as an overarching objective, a multifaceted approach to functional preservation may be the answer to this continued challenge. This review will examine these issues in depth in an effort to better understand postoperative functional outcomes with a focus on the younger, active breast reconstruction patient.

## INTRODUCTION

Mastectomy trends have recently been studied in depth, demonstrating that the rate of bilateral mastectomy is increasing, whereas unilateral mastectomy rates decrease.^1^ Much of these data come from The Nationwide Inpatient Sample, the largest all-payer inpatient care database in the United States. Data also suggest that immediate breast reconstruction rates are increasing, a trend that seems to be largely within the implant-based reconstructive modality.

This is due in large part to the increased utilization of contralateral prophylactic mastectomy and a slight rise in bilateral prophylactic procedures. One of the strongest factors associated with utilization of contralateral prophylactic procedures is young age, specifically women younger than 39 years.^2^ These patients, combined with other women younger than 50 years, make up the majority of bilateral prophylactic procedures. This substantial group of patients, with generally good prognoses and decades of life ahead, are of particular interest when it comes to optimizing physical function following breast cancer and reconstruction.

In the context of this rise in bilateral mastectomy rates, there has been an average increase of 5% per year in rates of immediate breast reconstruction.^1,3,4^ This seems to be driven largely by an increase in the use of implants as opposed to autologous reconstruction. Recent studies have found that free flap success rates approach 98% and implant-based success rates are in the mid 90s.^5,6^ Regardless of the modality, it is well accepted that breast reconstruction confers a significant psychosocial and aesthetic benefit for patients after mastectomy.^7^

The larger picture for both mastectomy and reconstruction is postoperative quality of life, a key component of which is function. To date, a number of studies have begun to look at the impact of reconstruction on form and function, yet few have looked at potential strategies for improving this key outcome. The purpose of this review was to critically examine the data with regard to functional outcomes following breast reconstruction and to challenge the plastic surgeon to think beyond the aesthetic result of the breast cancer treatment. We aim to present further approaches to optimize outcomes, as this is critical to the overall reconstructive outcome and thus particularly meaningful to the younger breast reconstruction patient.

## TOOLS TO ASSESS FUNCTION

Both objective and subjective tools are available and imperative in the overall assessment. The former relies on standardized measurements such as goniometry, dynamometry, video analysis of movement, and electromyography recordings to directly assess patient function after surgery, all of which provide raw data. Subjective tools on the other hand rely on the patient experience and perception of her condition.

Many such tools have been developed and validated specifically for breast cancer patients as well as for the upper limb in general. The BREAST-Q survey, which evaluates satisfaction and surgery-related quality of life following breast reconstruction, has been extensively utilized in plastic surgery to assess outcomes.^8^ It has become, in many ways, the gold standard for postreconstruction subjective evaluation. The Functional Assessment Cancer Treatment – Breast (FACT-B) is another questionnaire assessing multidimensional factors affecting quality of life following breast cancer treatment.^9^ The short form 36 (SF-36) focuses on both physical and mental health as components of quality of life, but is a more general questionnaire utilized in multiple areas of medicine and does not address the unique specifics of the postbreast reconstruction patient.^10^ Lastly, the Disabilities of the Arm, Shoulder, and Hand (DASH) emphasizes pain-related upper extremity disability in general and is validated across multiple fields.^11^ Subjective measures are fundamental to understanding how breast surgery affects the patient in a way that matters most to her.

## FUNCTION AFTER CANCER

Cancer patients often have functional deficits after treatment. The National Health and Nutrition Examination Survey found that in comparison with individuals with no history of cancer, both recent and long-term cancer survivors are more likely to report limitation in physical performance.^12^ Furthermore, data from the Nurses Health Study, which examined over 100,000 women, showed that female breast cancer survivors report a decline in functional health status after their breast cancer diagnosis, regardless of the cancer stage.^13^ These findings demonstrate the need to evaluate and understand how breast surgery affects function so that these sequelae can be prevented, addressed, and properly communicated to patients.

## THE FUNCTIONAL IMPACT OF BREAST SURGERY AND MASTECTOMY

As breast reconstruction inherently follows mastectomy, morbidities associated with the latter must be taken into account to fully understand the functional impact on patients. Studies suggest that breast surgery is associated with significant subjective and objective functional impairment^14,15^. These operations typically impact the upper quarter, with upper quadrant dysfunction (UQD) subsequently including pain, lymphedema, restricted mobility, impaired sensation, and strength.^14^ The most common types of UQD have been reported as pectoralis tightness at 3 and 6 months and lymphedema at 12 months, along with higher rates of rotator cuff disease long term.^15^

The long-term repercussions of upper limb dysfunction have also been examined. In one large Australian cohort, UQD affected over 50% of patients at 6 years postdiagnosis.^16^ Similarly, in a study using the FACT-B questionnaire as well as an objective exercise protocol evaluating upper body strength and endurance, a significant proportion of women experienced persistent functional deficits at 18 months postoperatively.^17^ The prevalence and persistence of UQD is particularly important, because higher levels of UQD seem to be associated with decreased quality of life,^18–21^ which is itself associated with decreased survival.^22^

Clinical circumstances and patient preference ultimately determine the surgical approach to breast cancer treatment, and there are notable differences in postoperative function among the various surgical options. Mastectomy is more likely to lead to UQD than breast conservation therapy,^23^ and even in the absence of self-reported pain, there seem to be altered motion patterns of the scapula on the side of the mastectomy. Although this has unclear functional significance, it does offer possible prognostic value and a role for physical therapy postmastectomy.^24^ Recent trends in lymph node evaluation also carry functional consequences, as UQD is more common in axillary lymph node dissection than in sentinel lymph node dissection.^25^

More invasive procedures lead to higher prevalence of dysfunction. This is the platform upon which breast reconstruction is performed, making it crucial for reconstructive techniques to account for functional impact on patients and to minimize this to the extent possible.

Three modalities of breast reconstruction are currently employed: tissue expander/implant-based reconstruction (E/I), autologous reconstruction using the patient’s own tissue, and a combination of these first 2 modalities. Research into the functional impact of reconstruction has however focused mainly on 3 areas: (1) function following E/I reconstruction; (2) function following latissimus dorsi (LD) reconstruction (which can be utilized as an autologous modality or more commonly in combination with E/I reconstruction); and (3) function following autologous reconstruction using abdominal tissue.

## FUNCTIONAL IMPACT OF IMPLANT-BASED RECONSTRUCTION

E/I-based reconstruction is currently the most common choice for postmastectomy reconstruction and in most cases involves manipulation of the pectoralis major muscle. One approach is total submuscular coverage, where tissue expander is placed in a plane beneath the pectoralis muscle medially and a portion of the serattus muscle laterally. In this technique, the major attachments of the pectoralis remain in place, but the muscle is attenuated and its force vectors altered over the course of expansion. Currently, there is no direct literature on how this technique affects function.

Another approach to E/I reconstruction is partial muscular coverage with release of the inferior and medial inferior insertions of the pectoralis with placement of a tissue expander or permanent implant under the muscle and the inferior portion of the prosthesis supported by biologic or absorbable mesh. At times, the inferior aspect of the prosthetic can be placed in the subcutaneous plane inferiorly. Several recent studies have assessed function following this release (Table 1) with the implant inferiorly being in a subcutaneous plane. De Haan et al.^26^ found the pectoralis muscle of the operated side to have a significant decrease in torque strength compared with the unoperated side. Although limited by the lack of a mastectomy-only comparison group, it does suggest functional loss in patients who undergo both mastectomy and partial muscular coverage implant.

**Table 1.**
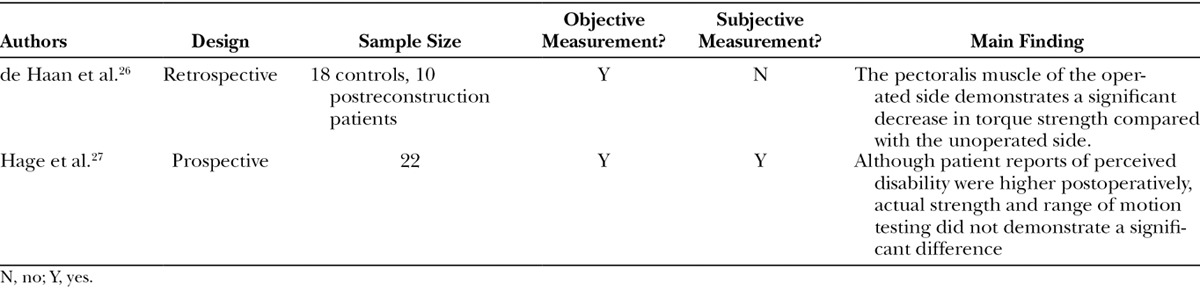
Literature on Function following Implant-Based Reconstruction

Several years later in 2014, Hage et al.^27^ compared preoperative and postoperative upper limb function in patients undergoing bilateral mastectomy and partial subpectoral implant. Although patient reports of perceived disability were higher postoperatively, actual strength and range of motion testing did not demonstrate a significant difference. In addition, patients produced greater electromyographic activity in the clavicular part of the pectoralis postoperatively, suggesting a compensatory functional change. This would further suggest that patient disability following mastectomy and partial subpectoral implant is not consequential for day-to-day function. No studies to date have examined function after prepectoral E/I placement.

Overall, the conclusions of these limited studies are unclear, with available data suggesting some functional changes following E/I reconstruction. More work is needed to accurately understand how it affects postmastectomy functional recovery, specifically in patients who undergo expander placement with total submuscular coverage. As this is the most common form of reconstruction, these data are needed for appropriate preoperative counseling of patients.

## FUNCTIONAL IMPACT OF LD RECONSTRUCTION

LD flaps are commonly used in for breast reconstruction in conjunction with E/I, especially in irradiated fields. Much work has been done on this approach, largely focusing on the donor-site morbidity (Table 2).

**Table 2.**
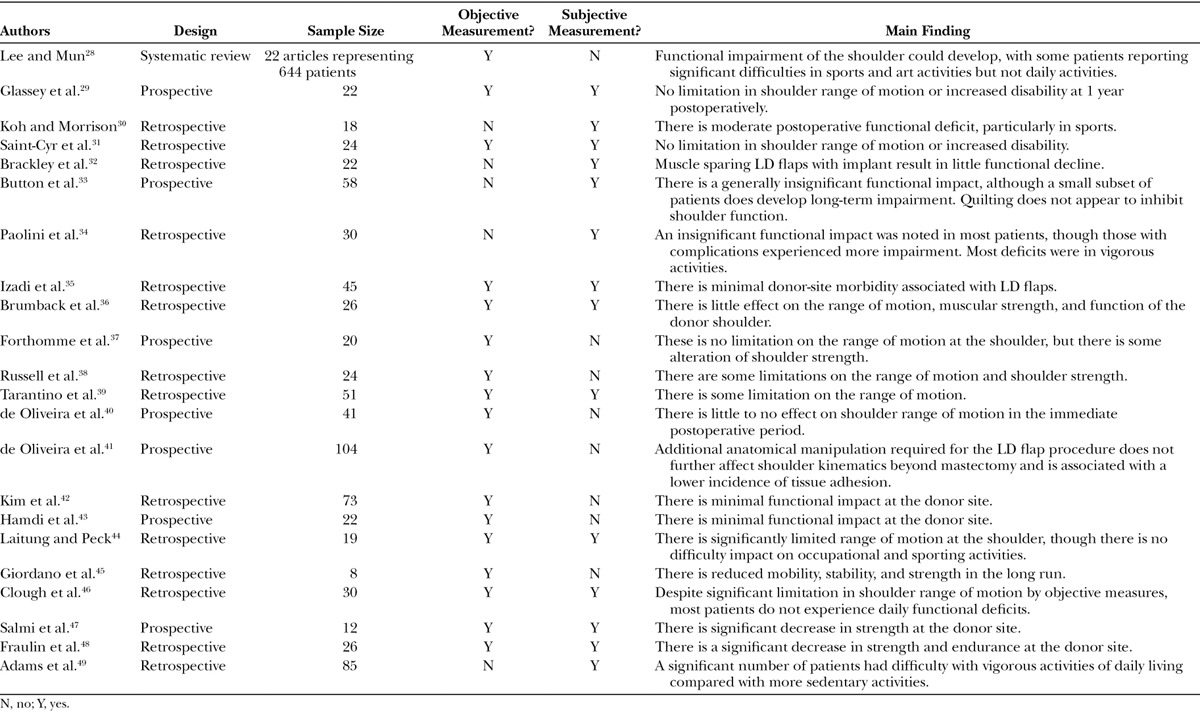
Literature on Function following LD Flap Reconstruction

A recent systemic review published in 2014 offers the highest level of evidence regarding the functional impact on donor-site morbidity following this procedure.^28^ This study found that overall, patients reported significant difficulties in sports and art activities but not daily activities. Seven studies presented data using the DASH questionnaire, with all scores reported to be less than 20 for daily activity (DASH scores range from 0 to 100, with 0 being no disability and 100 being the most severe disability).^29–35^ Reported DASH scores were more diverse for sports/art activities, ranging from 2.9 to 84.3 among studies. The review also examined numerous objective range of motion measurements among various studies, finding that 4 reported no range of motion (ROM) problems,^29,31,36,37^ 6 reported some,^38–43^ and 3 reported significant limitations.^44–46^ Lastly, 8 of the 12 studies reported significant shoulder strength limitations.^29,36–38,45–48^ From this review, it is difficult to conclude whether ROM and shoulder strength are substantially affected by LD flaps, but it does seem there is little perceptible disability at least in daily activities.

One recent prospective cohort study further focused on shoulder ROM after surgery.^41^ According to kinematic measurements 1 month after surgery, ROM was decreased by 30%. However, shoulder flexion and abduction capacity recovered to 5–10% lower than baseline over time. Of note, patients undergoing both mastectomy and LD reconstruction had slightly superior ROM scores than those undergoing only mastectomy, with immediate reconstruction being associated with decreased tissue adhesion at 12 months.

Corroborating the above studies’ conclusions, another retrospective cohort study found that functional arm morbidity following bilateral mastectomy and LD reconstruction generally improved over 1 year, with substantial dysfunction limited to certain subsets of activity.^49^ Specifically, a significant portion (44–73%) of patients had difficulties with vigorous activities of daily living, and 27–39% of patients reported moderate or worse difficult performing athletics. Additionally, another prospective study from 2015 demonstrated decreased DASH scores and SF-36 Physical Health Quality of life scores at 1 year, which did not return to baseline.^50^

The general findings of studies on LD reconstruction suggest that although some UQD may occur, most patients experience insignificant limitations on their day-to-day activities. Furthermore, those limitations seem to generally resolve with time. However, it is important to consider that nearly one-third of patients have reported difficulties in athletic function or in vigorous activities of daily living. Younger patients are more likely to engage in such physical stressors and may have a higher likelihood of experiencing these difficulties.

## FUNCTIONAL IMPACT OF ABDOMINALLY BASED AUTOLOGOUS RECONSTRUCTION

Abdominal tissue is considered by many to be the gold standard for postmastectomy breast reconstruction, although not all patients are candidates for this modality. Current modalities include deep inferior epigastric artery perforator (DIEP) flap, superficial inferior epigastric artery (SIEA) flap, pedicled transverse rectus abdominis myocutaneous (pTRAM) flap, and free TRAM (fTRAM) flap. Much of the focus has, understandably, been on the integrity and function of the abdominal wall donor site, but no consistent methodology, such as DASH for upper limb dysfunction, has emerged to date. The type of flap performed in autologous reconstruction often depends upon perfusion patterns as well as surgeon expertise in microsurgical techniques.

The highest level of evidence in the literature comes from a systematic review examining publications through 2007 comparing the various abdominal flaps^51^ (Table 3). This study found that patients receiving TRAM flaps were more likely to experience objective deficits in abdominal flexion and extension than those receiving perforator flaps. Examining studies using isokinetic dynamometry, this study found up to 23% trunk flexion deficit in pTRAM and up to 18% in fTRAM.^52^ Sit-up ability ranged from 27%^53^ to 71%^54^ for pTRAM and 47%^55^ to 82%^54^ for fTRAM. There were no significant differences between the 2 types of TRAM procedures, although not all studies differentiated degree of muscle sacrifice. Most importantly, except for those with bilateral TRAM procedures, the functional disadvantages of TRAM flaps decreased over time, with most women eventually returning to baseline function. Furthermore, although both TRAM modalities were associated with increased dysfunction over DIEP flaps, this discrepancy did not seem to translate to actual impairment of daily activity.

**Table 3.**
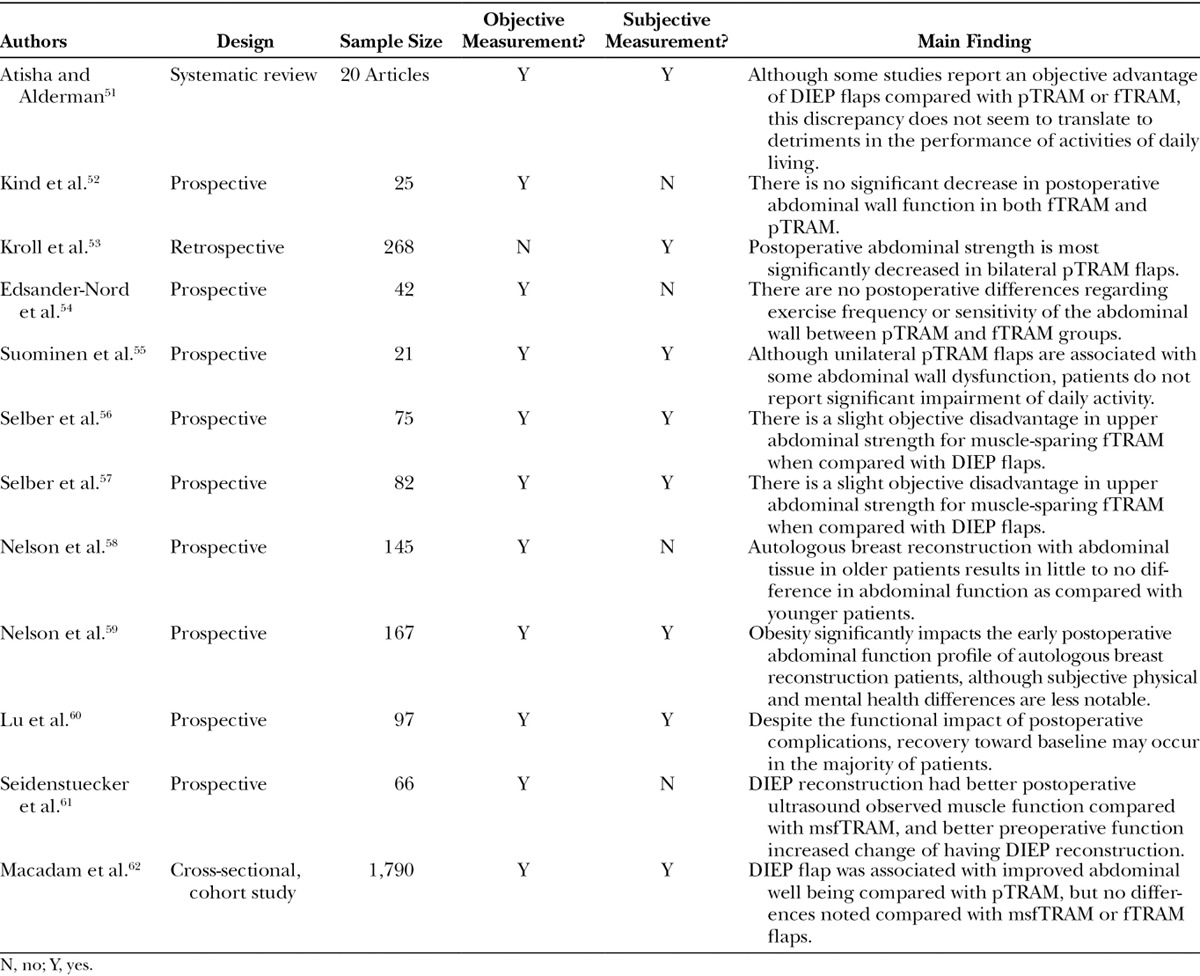
Recent Literature on Function After Abdominally Based Autologous Reconstruction

Some of the most focused recent work on abdominal wall function after autologous reconstruction has come from the University of Pennsylvania, examining objective and subjective function in just over 150 patients. The first study from this group noted a slight objective disadvantage in upper abdominal strength for muscle-sparing fTRAM when compared with DIEP flaps.^56^ Similar results were found when bilateral reconstruction outcomes were assessed using the same measurements.^57^ However, most importantly, these differences did not correlate to significant subjective declines in physical health as reported using the SF-36 questionnaire. This group then further examined the functional impact and found that age did not significantly impact functional outcome.^58^ However, postoperative early objective function significantly worsened in bilateral obese patients, although the same was not noted in subjective analysis.^59^ Importantly, postoperative complications were found to significantly impact early physical health, mental health, abdominal strength, and patient satisfaction. However, beyond 1 year, recovery toward baseline seems to occur in the majority of patients.^60^ Long-term (10-year follow-up) data (unpublished) further support a return to baseline function regardless of flap type.

Interestingly, another recent study also suggests that better preoperative rectus function can improve the likelihood of DIEP reconstruction,^61^ which could ultimately afford the patient with a more favorable functional profile in early postoperative recovery.

Despite some data suggesting objective differences in donor-site morbidity among the various types of abdominally based autologous reconstruction, patient subjective perception of function is not significantly different.^55,56,62^ However, the heterogeneity of surgical technique and patient population make this a difficult absolute conclusion to draw.

## COMPARISONS OF TECHNIQUES: PATIENT-REPORTED OUTCOMES

Several studies have examined patient-reported outcomes and specifically physical well-being in patients who underwent autologous tissue reconstruction compared with E/I reconstruction. One recent study from Memorial Sloan Kettering Cancer Center in 2014 suggested that patients who underwent I/E as well as autologous reconstruction had significantly higher Breast-Q physical well-being scale scores compared with patients who did not undergo reconstruction. Autologous patients however experienced significantly less chest and upper body morbidity than the I/E group.^63^ Early postoperative recovery was also recently examined utilizing the Mastectomy and Reconstruction Outcome Consortium study. In early recovery at 3 months, physical wellbeing had not returned to baseline in any modality. Chest and upper body physical morbidity were again significantly worse in TE/I patients compared with autologous reconstruction patients at this timepoint.^64^ Conversely, a study out of Michigan in 1995 found that TRAM flap recipients report more dissatisfaction with overall postoperative function compared with E/I.^65^ Another recent study utilizing the Mastectomy and Reconstruction Outcome Consortium study also demonstrated a significant difference in BREAST-Q physical function at 1 year across all modalities, but showed a return to baseline by 2 years, although this study did not differentiate by modality.^66^ Another multi-institutional study at a 2-year timepoint also found no significant differences in patient-reported outcome measures using several validated instruments (SF-36, FACT-B), but this time across modalities.^67^ With these somewhat conflicting findings, a definitive comparison of reconstruction modalities and their objective and subjective functional outcomes remains elusive.

## THE ROLE OF REHABILITATION

Regardless of the reconstructive modality, functional deficits are to be expected, especially early in the postoperative period. Minimizing such deficits and optimizing posttreatment function may depend not only on the surgical technique but also physical therapy. A recent systematic review looking at upper limb dysfunction following breast cancer treatment found that early structured exercise intervention may lead to significant improvement of shoulder range of motion, though an increase wound drainage volume and duration can be noted.^68^ Similarly, an observational prospective trial concluded that early assisted mobilization and home rehabilitation reduced the postoperative side effects and complications of breast surgery overall.^69^ Furthermore, it is notable that in 1 of the studies above which demonstrated no long-term functional deficit following LD flap reconstruction, patients were started on physical therapy 3 times a week for 4 weeks starting the first day postoperatively.^41^ These studies all begin to point to the potential advantageous role of early physical therapy in this cohort of patients, but research overall is lacking.^70^

Integration of postoperative function as part of breast surgery outcomes requires careful assessment both before surgery and at frequent intervals afterward. One proposed model proposes comprehensive preoperative objective and subjective functional testing, which makes it possible to accurately identify postoperative rehabilitation potential and need.^71^ Given the promising benefits of early exercise intervention for functional recovery, such a model would be helpful for ensuring that functional outcome is emphasized in delivering patient-centered care.

## CONCLUSIONS

Mastectomy with or without breast reconstruction can have functional ramifications. Regardless of the specific reconstructive modality, most objective measurements demonstrate functional deficits following surgery. Although not all deficits are subjectively perceived, it is important to consider functional impact as a key component of evaluating the overall outcome of breast surgery.

In reconstructive surgery, many conflicting goals often emerge including the aesthetic result, technical success, patient satisfaction, and functional impact. As rates of mastectomy and reconstruction increase, function is perhaps 1 of the most important considerations for long-term patient outcomes. For a patient who has decades of life ahead of her, a potential functional deficit could amount to decades of suboptimal function. The impact of this on quality of life and possibly survival is critical to keep in mind when choosing a reconstructive method.

A broader perspective on minimizing functional decline after breast surgery should not focus on only the surgical technique but also postoperative rehabilitation. Although not strictly a part of the surgeon’s purview, early, directed physical therapy could be an instrumental element in facilitating return to baseline function. With the patient’s optimal quality of life as an overarching objective, a multifaceted approach to functional preservation may be the answer to this continued challenge.
